# Encapsulation of Concentrated Solution Obtained by Block Freeze Concentration in Calcium Alginate and Corn Starch Calcium Alginate Hydrogel Beads

**DOI:** 10.3390/gels9050374

**Published:** 2023-05-01

**Authors:** Patricio Orellana-Palma, Loren Macias-Bu, Nailín Carvajal-Mena, Guillermo Petzold, Maria Guerra-Valle

**Affiliations:** 1 Departamento de Ingeniería en Alimentos, Facultad de Ingeniería, Campus Andrés Bello, Universidad de La Serena, Av. Raúl Bitrán 1305, La Serena 1720010, Chile; 2 Facultad de Ciencias Tecnológicas, Universidad Nacional de Agricultura, Catacamas 16201, Honduras; lmacias@unag.edu.hn; 3 Departamento de Ingeniería en Alimentos, Facultad de Ciencias de la Salud y de los Alimentos, Campus Fernando May, Universidad del Bío-Bío, Av. Andrés Bello 720, Chillán 3780000, Chile; nailin.carvajal1801@alumnos.ubiobio.cl (N.C.-M.); gpetzold@ubiobio.cl (G.P.); 4 Departamento de Nutrición y Dietética, Facultad de Ciencias Para el Cuidado de la Salud, Campus Concepción, Universidad San Sebastián, Lientur 1457, Concepción 4080871, Chile

**Keywords:** sucrose solution, gallic acid solution, block freeze concentration, encapsulation, hydrogel beads, in vitro digestion

## Abstract

A model (sucrose and gallic acid) solution was concentrated by block freeze concentration (BFC) at three centrifugation cycles, and the solutions were encapsulated in calcium alginate and corn starch calcium alginate hydrogel beads. Static and dynamic tests determined the rheological behavior, differential scanning calorimetry (DSC) and Fourier transform infrared spectroscopy (FTIR) established thermal and structural properties, and the release kinetics was evaluated under in vitro simulated digestion experiment. The highest efficiency encapsulation value was close to 96%. As the concentrated solution increased in terms of solutes and gallic acid, the solutions were fitted to the Herschel–Bulkley model. Moreover, from the second cycle, the solutions exhibited the highest values of storage modulus (G′) and loss modulus (G″), contributing to form a more stable encapsulation. The FTIR and DSC results demonstrated strong interactions between corn starch and alginate, establishing a good compatibility and stability in the bead formation. The kinetic release model under in vitro conditions was fitted to the Korsmeyer–Peppas model, demonstrating the significant stability of the model solutions inside the beads. Therefore, the present study proposes a clear and precise definition for the elaboration of liquid foods obtained by BFC and its incorporation inside an edible material that facilitates the controlled release in specific sites.

## 1. Introduction

Freeze concentration (FC), in comparison with traditional concentration technologies (evaporation) [1,2], is an emerging concentration technology that makes it possible to obtain liquid foods with a high concentration of solutes and a remarkable preservation and stabilization of valuable components such as polyphenols, vitamins, proteins, and minerals [3,4,5]. Hence, FC has been characterized as a concentration process at low temperatures, where, as the temperature of the liquid food decreases (below its freezing point, avoiding eutectic temperature), there is a phase separation by a counter diffusion phenomenon. In this phase separation, as the temperature decreases, the ice crystals expel the impurities (solutes, vitamins, and bioactive compounds, among others) towards their walls. Thus, it is possible to separate the concentrated (liquid sample) from the ice crystals (porous solid fraction) [6].

Therefore, FC prolongs the shelf life of different liquid foods such as juice, coffee, tea, and milk, among others, providing a better availability of various types of fruits and vegetables throughout the year beyond the normal seasonality [7]. However, there are still challenges that must be appropriately addressed to protect the properties of the concentrated solutions obtained by FC against external factors such as water, light, oxygen, pH, enzymatic degradation, and temperature, since even the concentrated solutions are chemically or physically instable, with refrigeration being an excellent alternative to maintain their properties, albeit at a high economic cost [8]. Moreover, the concentrated solutions can be degraded in gastrointestinal conditions, and thus, the bioactive components are not absorbed in body [9]. Therefore, it is very important to find technologies that protect various components from environmental factors, and in turn, it facilitates a targeted delivery of concentrated components in specific sites, e.g., in the intestinal tract [10].

Hence, for many years, the encapsulation process has been developed and improved to retain, protect, and liberate valuable bioactive substances by their entrapment in the coating materials [11]. In this sense, alginate (GRAS, Generally Recognized as Safe) has been the most widely used for encapsulation of foods [12,13]. However, calcium alginate hydrogel beads have high syneresis (release of sample from the gel matrix) due to the empty spaces (pores) in the walls, and therefore, the efficiency of the encapsulation must be reinforced with the addition of corn starch for filling the pores, reducing the size of the pores, and preventing the output of sample encapsulated to the outside [14].

Various studies have shown the beneficial effect of encapsulation using calcium alginate hydrogel beads in the protection of bioactive compounds, for example, polyphenols from chokeberry extracts [15], anthocyanins from hibiscus extracts (the beads were added to a yogurt sample, obtaining a high sensory perception) [16], stevia extracts [17], olive leaf extracts [18], and *Moringa oleifera* extract [19], among others. In the same way, in the last year, our group has successfully explored the potential to form stable calcium alginate hydrogel beads filled with model solution (mix of sucrose and gallic acid) obtained by block FC (BFC), where loading capacity, particles size, shape, water activity, moisture content, and bulk density were studied [20]. Nevertheless, there is still a significant research gap, and future innovative studies on the rheological and thermal behaviors, among other interesting properties, and post-encapsulation of concentrated solutions acquired by BFC are required to obtain scientific knowledge that makes it possible to predict the kinetics of release of polyphenols encapsulated under simulated digestion conditions.

Therefore, the present work is a continuation on the study of sucrose/gallic acid solution obtained by BFC encapsulated in calcium alginate (Alg) hydrogel beads and corn starch-calcium alginate (CSAlg) hydrogel beads [20], where the innovation is emphasized on the new information obtained in the analysis of the rheological properties, thermal characteristics, and the different behavior patterns inside an artificial digestive system. Additionally, this work offers information for the elaboration of future foods with a high content of polyphenols inside an edible material that allows the controlled release of bioactive compounds in specific sites.

## 2. Results and Discussion

### 2.1. EE%

**Figure 1:**
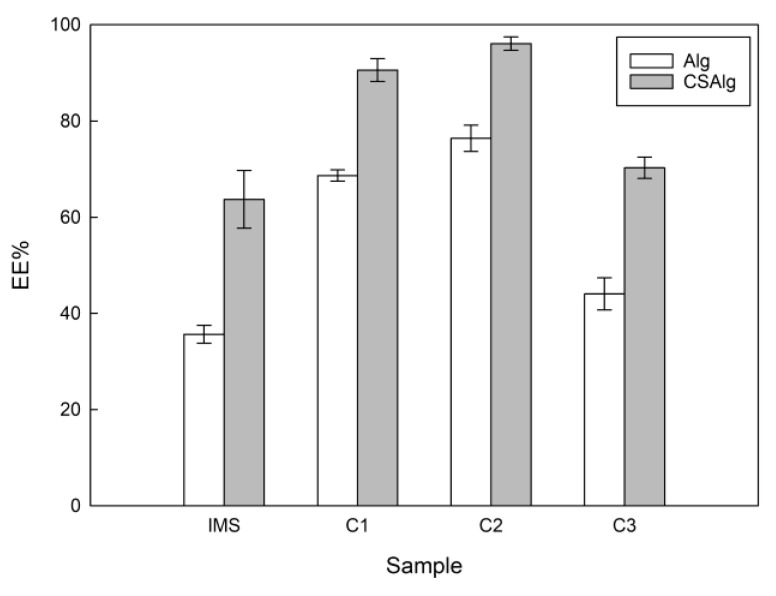
Effects of concentration of sucrose/gallic acid solution on the encapsulation efficiency.

Figure 1 shows the EE% of the CSAlg and Alg hydrogel beads filled with initial model solution (IMS), concentrate from cycle 1 (C1), concentrate from cycle 2 (C2), and concentrate from cycle 3 (C3) solutions.

In the present study, it is important to mention that our research group studied the encapsulation efficiency of the different concentrations of sucrose/gallic acid solution (called IMS, C1, C2, and C3) and used the best results in terms of concentration of CSAlg (corn starch at 2% (*w*/*w*) and sodium alginate at 2% (*w*/*w*)) from a previous study [20]; in addition, we added only sodium alginate (2% *w*/*w*) as control (Alg) (the procedure is indicated in Section 4.4). Through this method, the results revealed statistically significant differences between each concentrated solution (IMS vs. C1 vs. C2 vs. C3) and materials (CSAlg vs. Alg) to encapsulate the solutions. Furthermore, the lowest EE% values were obtained in hydrogel beads filled with IMS, and the highest EE% values was reached in hydrogel beads filled with C2. Hence, for Alg hydrogel beads filled with IMS, C1, C2, and C3, the EE% values were close to 36%, 69%, 76%, and 44%, respectively. Instead, for CSAlg hydrogel beads, the EE% results were higher than Alg hydrogel beads samples, with 64%, 91%, 96%, and 70%, filled with IMS, C1, C2, and C3, respectively.

Specifically, the highest EE% value in CSAlg/C2 (96%) was similar to that reported previously in the literature [15,20]. The EE% result can be explained by various factors, the behavior of the C2 solution being an important point, since the solutions obtained by BFC have been recognized for their high concentration of solutes [6,21]. Hence, the solutes can contribute as a unifier, allowing the adhesion of corn starch to sites still uncovered (nanopores). Therefore, current knowledge of the properties of the solutions clarifies the best options for a correct encapsulation process through ionic gelation [22].

**Figure 2:**
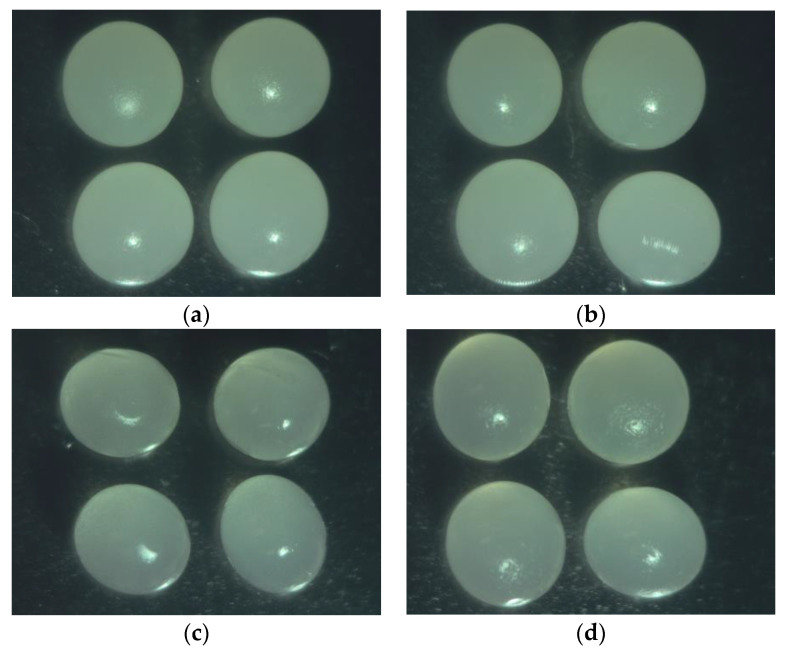
Digital microphotographs of CSAlg hydrogel beads filled with solution: (**a**) IMS; (**b**) C1; (**c**) C2; (**d**) C3.

Moreover, our previous results indicated that calcium alginate at 2% (*w*/*w*) produces better cross-linking of molecules than other concentrations. Thus, the porous structure (empty spaces) in the walls has a small size due to the formation of thick connections [23], and later, the full encapsulation process was achieved by adding corn starch solution at 2% (*w*/*w*), since the corn starch fills the pores due to its granulated shape that facilitates the production of a block in the outlet of any solution to the outside [24]. Hence, the hydrogel bead matrix is strengthened in terms of efficiency, viscosity, spherical shape, and size (Figure 2), among other encapsulation factors [25].

Additionally, this correct concentration between sodium alginate and corn starch for the formation of hydrogel beads can be explained through the electrostatic stability, since Feltre et al. [26], via zeta potential measurements, indicated that a CSAlg mixture showed stable electrostatic stability due to the high electronegative zeta potentials (values > 65 mV), where alginate promotes high negative charge in the mixture, contributing stability by decreasing the interfacial tension and increasing steric forces, and where corn starch decreases the electronegativity of the mixture, and thus, it is possible to equilibrate the system. Moreover, the CSAlg hydrogel beads filled with solution showed an increase in swelling ratios, since the samples increase in size by retaining the concentrated samples, and its behavior can be maintained for a considerable time due to the addition of corn starch. Wang et al. [27] indicated that the beads maintained a swelling equilibrium from 30 min to 24 h. This phenomenon can be explained by the process of absorption and retention of water from the concentrated sample within their gel structure, and thus, the stable hydrogen bonds can be formed among the penetrated water from liquid sample, corn starch, and alginate [28].

Furthermore, the size of the CSAlg hydrogel beads filled with solution decreased significantly, with diameter values close to 3.08 ± 0.02 mm, 2.84 ± 0.02 mm, 2.72 ± 0.03 mm, and 2.66 ± 0.02 mm, for CSAlg/IMS, CSAlg/C1, CSAlg/C2, and CSAlg/C3, respectively. This behavior can relate to the interaction between the mix of alginate/cornstarch/sucrose/gallic acid, since IMS has high solubility, and it does not have a great influence in the interstitial empty spaces. At the same time, from C1 to C3, the diameter decreased due to the concentrated sample has low solubility, achieving a greater interaction with the molecular bond interactions, causing a closure of the interstitial empty spaces [20].

In addition, our findings are in line with prior research, since the EE% result was higher than the encapsulation of polyphenols extracted from rose hips, with EE% value close to 90.7%, where the encapsulation process was achieved with sodium alginate at 2% (*w*/*v*), without incorporation of corn starch solution [29]. Similarly, our result was higher than the encapsulation of extract from *Mesona chinensis* benth, with EE% value of approximately 55%, with a concentration of sodium alginate at 1.8% (*w*/*v*) [30], and in the same way, our EE% value was higher than the encapsulation of extract from yerba mate (*Ilex paraguariensis*) [23], with an EE% value close to 65%, using calcium-alginate at 3% (*w*/*w*) and corn starch at 2% (*w*/*w*). Therefore, these significant differences in EE% values indicate that the type and concentration of encapsulating material (sodium alginate at 2.0% (*w*/*w*), calcium chloride at 2.0% (*w*/*v*), and corn starch solution at 2.0% (*w*/*v*)), and in turn, the concentration of the solutes, facilitate an excellent encapsulation of liquid samples concentrated by a novel low temperature technology.

### 2.2. Rheological Behavior

**Figure 3:**
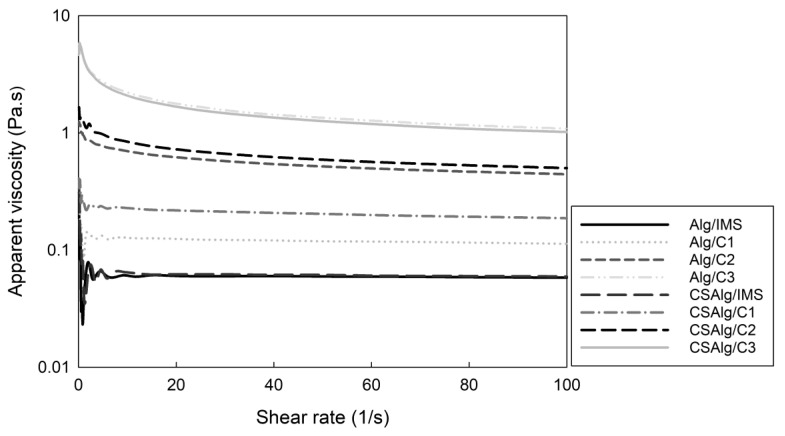
Apparent viscosity versus shear rate of the liquid mixtures.

Figure 3 shows the apparent viscosity versus shear rate for the mixture of Alg with the liquid samples and the mixture of CSAlg with the liquid samples.

The determination of the rheological properties provides insight into the different effects of the BFC process on the viscosity and molecular interactions between the mixture of Alg/IMS, Alg/C1, Alg/C2, and Alg/C3 solutions and CSAlg/IMS, CSAlg/C1, CSAlg/C2, and CSAlg/C3 solutions. Visually, the sodium alginate solution presented a translucent yellow color, while corn starch sodium alginate hydrogel solution formed a uniform opaque white color.

In general, Figure 3 had similar behavior, since Alg/IMS and CSAlg/IMS solutions had the lowest apparent viscosity in comparison to the other mixed solutions. At the same time, Alg/C3 and CSAlg/C3 had a contrary behavior, whereby Alg/C3 and CSAlg/C3 solutions presented the highest apparent viscosity, and this can relate to the concentrated sample, since C3 presents a higher viscosity than the other liquid samples, due to the high total soluble solids content and high gallic acid equivalent (GAE) concentration: IMS with 15.0 °Brix and 1000 mg GAE/L, C1 with 27.7 °Brix and 2117 mg GAE/L, C2 with 40.7 °Brix and 8309 mg GAE/L, and C3 with 49.5 °Brix and 11,100 mg GAE/L. These values were obtained from a previous study by our research group [20]; i.e., as the solutes and GAE solutions became more concentrated, it increased the viscosity of concentrated solutions. Therefore, the application of the BFC process in a liquid sample allows us to obtain a high total soluble solids content and high gallic acid equivalent concentration, and hence, it produces a final liquid sample with high viscosity [1,2,8].

Additionally, for all samples, at a low shear rate (between 0 and 20 s^−1^), the apparent viscosity of the solutions shows a decreasing trend with an increase in shear velocity, indicating that the mixtures had a pseudoplastic fluid and shear thinning behavior, while at high shear rate (>20 s^−1^), the apparent viscosity presented no significant differences as the shear rate increased; i.e., the solutions had a continuous behavior, without an increase or decrease in apparent viscosity with an increase in the shear rate, providing a transition from the non-Newtonian fluid at a low shear rate to Newtonian fluid at a high shear rate [31]. Zhao et al. [32] observed a similar behavior, since the results showed that the distribution of alginate chain lengths had a non-Newtonian behavior, where short chains are oriented more in the direction of shear stress than long chains. Thus, the higher the shear stress, the more the shorter chains are oriented, thus decreasing the viscosity in the solutions.

In particular, CSAlg mixed with any solution showed a higher apparent viscosity than Alg mixed with any solution (Table S1, Supplementary Materials), indicating that the incorporation of corn starch into the sodium alginate solutions resulted in an increase in the apparent viscosity due to the reinforcement of the interactions between the two polymers through electrostatic intermolecular repulsion, and in turn, the expansion of polysaccharide chains promoted the cross-linking of the chains [33], forming a more viscous solution in comparison to the samples without corn starch. A similar behavior was observed by Jaster et al. [34] and Casas-Forero et al. [35], who studied the incorporation of a concentrate obtained by BFC technology as an ingredient to produce yogurt and commercial hydrogels, respectively, where the rheological results indicated that the viscosity was decreased when the ratio of the concentrate was increased in the elaboration of the samples.

An important point to mention in the present study is that we determined the behavior of the solutions prior to the encapsulation process, since the amount of solution that passes through the needles in the extrusion process depends on the viscosity of the solution and the diameter of the needles. A hydrogel solution with high viscosity results in beads with a wall thickness more significant than the usually used, affecting the amount to be encapsulated, and different properties of the beads such as the resistance to external media and thermal properties, among others. Therefore, the nature and composition of the encapsulation solutions determine the viscosity behavior and, thus, the wall thickness of the beads obtained [36]. In the same way, Bhujbal et al. [37] reported that a minimum viscosity in the encapsulation solution of 4 cP (0.004 Pa·s) is necessary for spherical beads to form. Therefore, hydrogel solutions for encapsulation with low viscosities are associated with irregular fragments in the beads called satellites, where many broken beads are observed due to the lack of protection [38]. Meanwhile, the solutions with high viscosity are associated with undesired shapes and the formation of tails, where the generated droplets increase in size, since the coaxial air flow requires high flow rates to cut the droplet. Therefore, the hydrogel solutions used for encapsulation must exhibit optimal viscosity to produce beads with optimal shape and stable geometry [37]. Thus, as observed in the present study, the encapsulation solution obtained from the CSAlg with C2 shows medium viscosity, and in turn, it presents the best encapsulation efficiency (Figure 1) and the most spherical shape (Figure 2).

**Figure 4:**
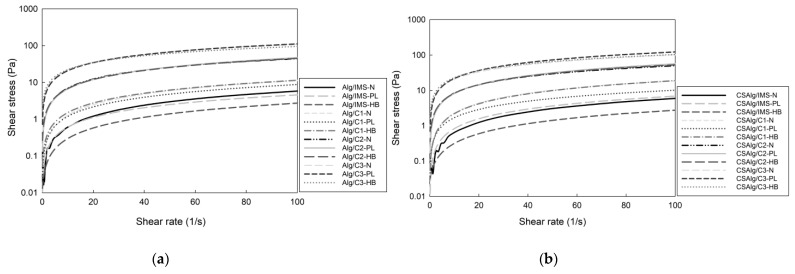
The fitting curve of the Newtonian model, the Power law model, and the Herschel–Bulkley model of hydrogel beads: (**a**) Alg with liquid samples; (**b**) CSAlg with liquid samples. Alg: sodium alginate solution; CSAlg: corn starch sodium alginate solution; IMS: initial model solution; C1: concentrate from cycle 1; C2: concentrate from cycle 2; C3: concentrate from cycle 3; N: Newtonian model; PL: Power law model; HB: Herschel–Bulkley model.

In the study, the flow curves (Figure 4a,b) were fitted with the Newtonian model, the Power Law model, and the Herschel–Bulkley model, highlighting that these models were chosen because they are the most used for concentrated solutions of food polymers.

Moreover, the choice of the models was made based on the results reported by other authors, evidencing the use of different polymeric solutions for the formation of hydrogel beads, for example, cashew gum for the encapsulation of oils [39], hydrogel solutions from the liquid core of barberry (*Berberis vulgaris*) [40], and maltodextrin as a wall material for the encapsulation of an extract of *Indigofera tinctoria* [41]. The study of Tavares et al. [42] on rheological trends in the encapsulation of bioactive compounds from essential oils was also considered, where it was found that the Power Law and Herschel–Bulkley models are the best fit in most cases.

Table 1 indicates the parameters obtained from data fitting to the Newtonian, Power Law, and Herschel–Bulkley models.

For the Newtonian model, the coefficients of determination (R^2^) varied in the range of 0.69–0.84, indicating a low dependence of the apparent viscosity with shear rate of the liquid samples. Therefore, this model was not considered for further analysis.

For Power Law and Herschel–Bulkley models, the R^2^ values varied in the range of 0.96–0.99, indicating the high dependence of the apparent viscosity with shear rate of the liquid samples. Furthermore, the flow behavior index (η) of the samples tended to decrease when a more concentrated solution was added, which is equivalent to a solution with high total soluble solids content and gallic acid solution, provoking the breaking of hydrogen bonds between the networks, leading to the unfolding of polymer chains of the hydrogels samples. In addition, all models showed η values less than 1, thus corroborating, as mentioned above, that the mixtures exhibited pseudoplastic (shear-thinning) behavior [43]. In the same way, the yield strength (τ_0_) values ranged from 0.01 to 3.99 Pa, indicating that low stress levels are required to initiate the flow of the solutions, and in turn, a solution with more solutes needs more stress for its mobility. Moreover, the consistency index (K) of the liquid samples significantly increased when a more concentrated solution was added in the Alg or CSAlg solution, and it indicated that the viscosity increased with an increase in the total soluble solids content and gallic acid solution obtained by BFC. This may be because as the solutes and gallic acid concentration increased, the interaction between the molecules and the polymers of the solutions increased, leading to a strengthening of the relative molecular motion [44]. Therefore, the results from the model parameters indicate that the liquid solutions can be easily extruded, and a continuous flow can be achieved at low pressures.

**Figure 5:**
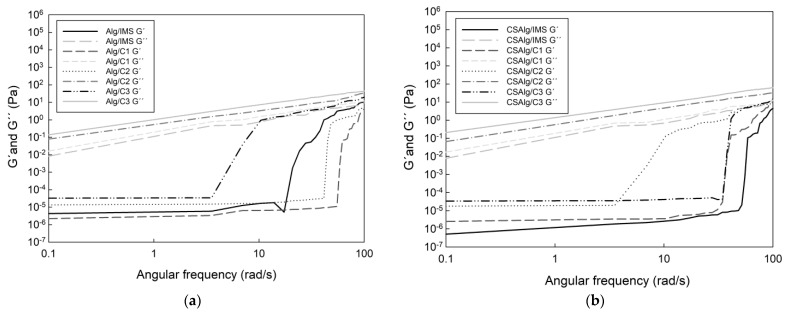
Storage (G′) and loss (G″) moduli of hydrogel solutions: (**a**) Alg with liquid samples (Table S2); (**b**) CSAlg with liquid samples (Table S3).

The changes in the storage (G′) modulus and loss (G″) modulus of mixed samples as a function of angular frequency are shown in Figure 5 (Tables S2 and S3, Supplementary Materials).

In all cases, the G′ modulus and G″ modulus increased with the angular frequency, where the values of G″ was higher than G′, indicating that the solutions presented predominance of the viscous component over the elastic one, verifying the typical behavior of liquid solutions [45]. Additionally, both G′ and G″ behaviors increased with an increase in concentration solutions, while the CSAlg with liquid solutions presented higher values of viscoelastic parameters than those containing only Alg with liquid solution. The solutions (CSAlg or Alg solutions) from the second BFC cycle exhibited high G′ and G″ values, which contributes to an increase in the viscosity of the solutions, and thus, it forms a more stable encapsulation process. Thus, the storage modulus and loss modulus were the highest in the hydrogel sample with C3, followed by the hydrogel sample with C2, hydrogel sample with C1, and hydrogel sample with IMS. Therefore, the rheological behavior shows that the concentrations of corn starch, calcium chloride, and sodium alginate present suitability and good flexibility to be used as a wall material, and in turn, it presents excellent mechanical strength and highly efficient encapsulation [46].

The effects in the incorporation of the concentrate obtained by BFC in hydrogel samples had similar results in terms of the G′ modulus and G″ modulus in comparison with other studies, since Casas-Forero et al. [35] used concentrated blueberry juice obtained by BFC as an ingredient in commercial gelatin gel, aerated gelatin gel, gummy, and aerated gummy hydrogels, and the samples presented an increase in G′ modulus and G″ modulus with the angular frequency. This can be explained by the hydrogel matrices, since a good concentration of hydrogel can retain liquid solutions between the interstitial spaces, and thus, the final samples present an excellent structure for the elaboration of commercial and edible hydrogels.

### 2.3. Fourier Transform Infrared-Attenuated Total Reflectance (FTIR-ATR) Spectroscopy Analyses

**Figure 6:**
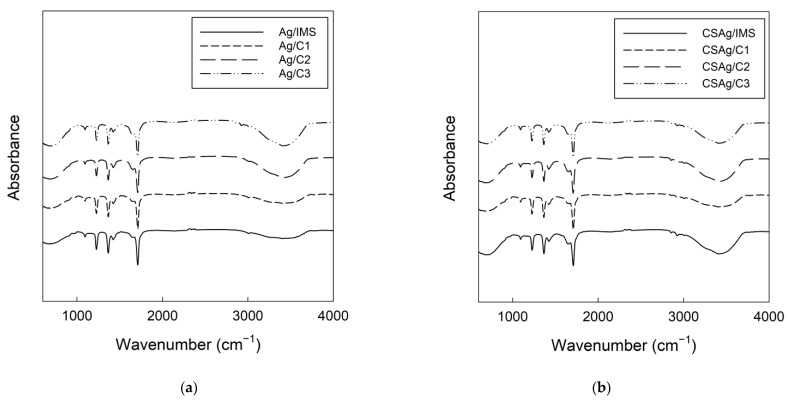
FTIR spectra of hydrogel beads: (**a**) Alg with liquid samples; (**b**) CSAlg with liquid samples.

Spectral data for Alg (Figure 5a) and CSAlg (Figure 5b) hydrogel beads filled with IMS, C1, C2, and C3 solutions are depicted in Figure 6.

In general, it is possible to see the same specific peaks in all samples, and as the solutes and GAE solutions become more concentrated, it is possible to denote certain peaks increased. Firstly, the peak at 1026 cm^−1^ is typical of polysaccharides or polysaccharides-like substances; in addition, it is composed of overlapping bands with a peak located at 1047 cm^−1^, which is associated with the formation of a more organized chain of polysaccharides or polysaccharides-like substances. Moreover, it is possible to appreciate a peak at 1022 cm^−1^, and it represents the amorphous structures and another peak at 1000 cm^−1^, which is linked to the hydrated structures [47]. The peak at 1155 cm^−1^ was attributed to the -CO stretching of the pyranose ring, and its intensity increased with the addition of concentrate solution from each BFC cycle; a similar trend was found in the peak at 1078 cm^−1^, which relates to the -C(1)-H bending of the corn starch glycoside ring [48]. The samples showed a prominent peak at 1411 cm^−1^, and its intensity gradually decreased with an increase in the addition of the concentrate solution, while the opposite occurred with non-starch solutions (Figure 4a), which relates to the symmetric stretching vibration of the -COO [49]. The spectrum of native corn starch (Figure 4b) showed a peak at 1640 cm^−1^, which can relate to the asymmetric stretching vibrations of the -COO, and this peak was shifted in all beads to around 1590 cm^−1^ [50]. At 1336 cm^−1^ and 2926 cm^−1^, it is possible to denote a characteristic peak of corn starch, which relates to the stretching of asymmetric -CH_2_ bonds [51]. For all samples, peaks between 3289 cm^−1^ and 3316 cm^−1^ were observed, which relate to the stretching of hydroxyl (OH) groups due to the formation of hydrogen bonds [50]. Hence, these peaks present a visible intensity in the initial solutions due to the high content of water, corresponding to mainly polysaccharides, and later, with the addition of concentrate solutions, the peaks present a high intensity [52]. Furthermore, strong interactions were established between corn starch and calcium alginate, demonstrating a good compatibility and stability for the formation of hydrogel beads.

### 2.4. Thermal Properties of Samples

**Figure 7:**
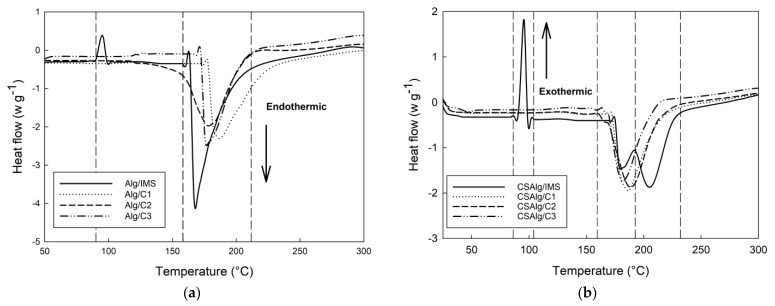
DSC thermograms of hydrogel beads: (**a**) Alg with liquid samples; (**b**) CSAlg with liquid samples.

Figure 7 depicts the DSC thermograms of the Alg (Figure 6a) and CSAlg (Figure 6b) hydrogel beads filled with IMS, C1, C2, and C3 solutions.

All samples showed a broad endothermic peak, but it was higher for Alg with IMS than the other samples. Additionally, the samples presented hydroxyl and carboxylate groups, which form intra- and intermolecular hydrogen bonds [48]. The total enthalpy and the temperatures of the peaks showed statistically significant differences between the samples (Table 2).

The hydrogel beads with IMS showed a first peak between 95 °C and 96 °C, and this may relate to the vaporization of the free water molecules contained in the polymeric network [53]. Likewise, the highest weight loss occurs in the first peak due to the removal of structural water and the desorption of water physically absorbed by the polymeric network [54]. Meanwhile, with the addition of corn starch (Figure 5b), it can be observed a significant increase in the enthalpy value from 142.29 mJ/g to 508.28 mJ/g, suggesting that the presence of corn starch increases the cohesive forces and physical integrity of the composite polymeric network [48].

The degradation of all beads occurred at values from 165.79 °C to 187.58 °C due to the cleavage of -CH bonds and -COC glycosidic bonds in the main chains [55]. The location of the second peak showed an increase in temperature with the addition of corn starch in the hydrogel samples with solutions. Furthermore, it can be observed that the enthalpy of the second peak decreased, reflecting the compatibility of both biopolymers (alginate and corn starch). Therefore, the reduction in enthalpy reflects the fact that corn starch forms weaker polymeric networks than alginate [48].

CSAlg/IMS beads presented a peak at 192.08 °C (ΔH = 264.44 mJ/g), and this can be attributed to the fact that adding alginate to a corn starch matrix requires higher temperatures to gelatinize the semi-crystalline starch structure, limiting the swelling of starch granules because both polymers compete to absorb water [55]. Similar results were reported by Feltre et al. [50], where microcapsules constituted by corn starch-alginate showed an increase in the temperature and ΔH of corn starch. On the other hand, the presence of this third peak can also be attributed to the lack of interaction between the bioactive compound (GAE) and the encapsulation matrix due to a lower presence of total soluble solids content [56].

### 2.5. Gallic Acid Kinetic Release under Gastric Conditions

Gallic acid solution released under simulated gastric conditions was evaluated according to the zero-order, first-order, and Korsmeyer–Peppas models. However, for the zero-order and first-order models, neither the release coefficient nor the exponent indicative of the release mechanism could be determined, since the release of gallic acid from the bead does not follow a linear pattern. Therefore, the data were fitted to the Korsmeyer–Peppas model (Table 3).

For k, the values presented statistically significant differences between treatments for each encapsulation method, where, in general, Alg samples presented higher k values than CSAlg samples; in addition, between all samples, the highest values were obtained with the solution C3, indicating that there was a higher diffusion in the Alg hydrogel beads into the medium than in the CSAlg samples [57].

For n, the values are less than 0.5 for all samples, ranging from 0.14 to 0.32, and therefore, the release of gallic acid is governed by a diffusion behavior [58,59], meaning that the hydrogel beads follow a pseudo-Fickian diffusion mechanism [60], and in turn, it indicates that the solution diffusion is greater than the relaxation process of the polymeric chain [61].

For R^2^, the values obtained were higher than 0.95, except for Alg/C3, with R^2^ value close 0.88, indicating a quite accurate model (from 0.70 to 0.90), since Valinger et al. [62] explicated that R^2^ values above 0.90 are a good-fitting model.

## 3. Conclusions

This study provided new information following the analysis of the rheological properties, thermal characteristics, and the different behavior patterns inside an artificial digestive system (in vitro study) of model solutions obtained by the BFC process inside hydrogel beads composed of sodium alginate at 2% (*w*/*w*) and corn starch at 2% (*w*/*w*). The flow behavior index, according to the Herschel–Bulkley model, showed values below η < 1 for the samples, which is indicative of non-Newtonian behavior. The storage modulus (G′) and loss modulus (G″) increased with the angular frequency, highlighting that the values of G″ were higher than the values of G′, indicating that the solutions presented the predominance of the viscous component over the elastic one, verifying the typical behavior of liquid solutions. All solutions present hydroxyl and carboxylate groups, forming intra- and intermolecular hydrogen bonds, which are responsible for the transition peak thermograms. The addition of corn starch showed values from 165.79 to 187.58 °C in DSC thermograms, indicating that corn starch chains increased the cohesive strength and physical integrity of the polymeric network. The high-intensity peaks found in calcium alginate hydrogel samples were also observed in the spectra of the corn starch calcium alginate hydrogel samples, although the location of these peaks shifted, reflecting the interactions between the two compounds. The kinetic release under gastric conditions values indicate that the release of sucrose and gallic acid was governed by diffusion, making that combination ideal for compounds that require a slow release and are suitable for wall material, with excellent flexibility and the mechanical strength necessary to obtain an efficient encapsulation.

## 4. Materials and Methods

### 4.1. Chemical Reagents

Sodium alginate, sucrose, calcium chloride (CaCl_2_), sodium carbonate (Na_2_CO_3_), calcium chloride dihydrate (CaCl_2_(H_2_O)_2_), sodium hydroxide (NaOH), sodium citrate (Na_3_C_6_H_5_O_7_(H_2_O)_2_), hydrochloric acid (HCl), potassium chloride (KCl), potassium dihydrogen phosphate (KH_2_PO_4_), sodium acetate (CH_3_COONa), sodium chloride (NaCl), magnesium chloride hexahydrate (MgCl_2_(H_2_O)_6_), ammonium carbonate (NH_4_)_2_CO_3_, gallic acid (GA), Folin–Ciocalteu regent, and ethanol were purchased from Merck (Merck, Darmstadt, Germany). Ultrapure water (18 Ω) and deionized water were obtained from a Milli-Q^®^ system (Thermo Fisher Scientific, Waltham, MA, USA).

### 4.2. Preparation of Concentrated Solution Obtained by BFC

**Figure 8:**
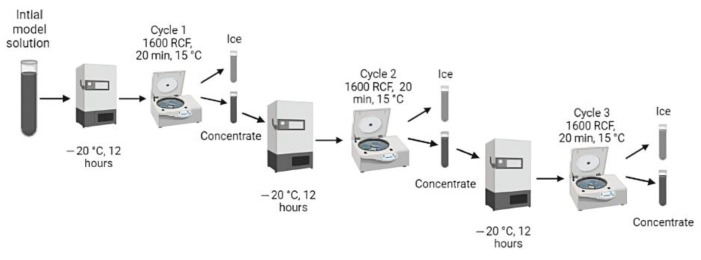
Scheme of centrifugal-assisted BFC process over three cycles.

The initial model solution (IMS) was prepared according to our previous work [63], where sucrose (15 °Brix) and GA (1000 mg/L) were mixed, and thus, IMS was subjected to three BFC cycles assisted by centrifugation as external force (Figure 8).

Hence, 45 mL of mixture solution was placed in centrifuge tubes covered with thermal-insulating foam (the upper part of the tubes was not covered with foam), and thus, the freezing step (overnight at −20 °C) was made through axial direction in a vertical static freezer (280, M and S Consul, Sao Paulo, Brazil). Therefore, the concentrated solution was extracted from the ice fraction using a centrifuge (Eppendorf 5430R, Hamburg, Germany) at 15 °C for 20 min at 1600 RCF (4000 rpm), and its process is equivalent to the cycle 1. Thus, the concentrated solution from C1 was used as a feed solution for the next cycles using the same procedure described above (centrifuge tubes with foam, freezing step, and centrifugation process). Thus, at the end of the centrifugal-assisted BFC process, one model solution and three concentrated samples were obtained.

### 4.3. Encapsulation of Concentrated Solutions Obtained by BFC

**Figure 9:**
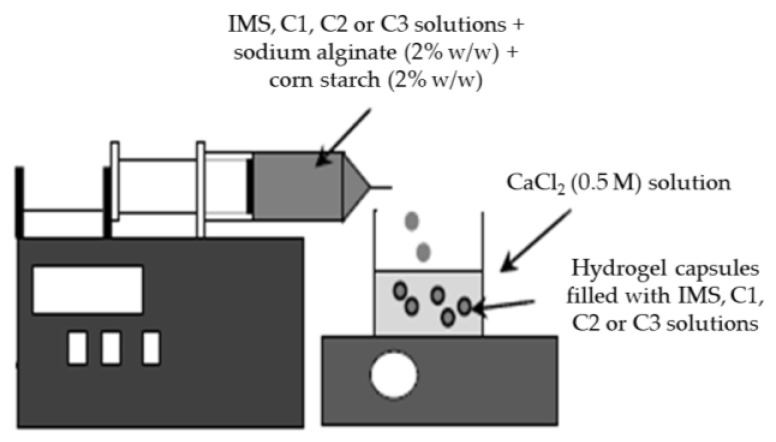
Scheme of encapsulation of concentrated solutions via ionic gelation technique.

The encapsulation process of liquid samples was prepared through ionic gelation technique, as previously described by Guerra-Valle et al. [20], where the results indicated that the best combination to encapsulate IMS and concentrated solutions obtained by BFC was corn starch at 2% (*w*/*w*) and sodium alginate at 2% (*w*/*w*) (called CSAlg); in addition, we have added a control, with only sodium alginate at 2% (*w*/*w*) (called Alg), to compare with the best combination (CSAlg). Specifically, sodium alginate and cornstarch powder was dissolved into distilled water, and later, the solution was subjected to constant agitation using a magnetic stirrer (RSM-14 HP, ProfiLab24 GmbH, Berlin, Germany) at 40 °C, and in the same way, CaCl_2_ (0.5 M) powder was dissolved into distilled water with constant agitation using a magnetic stirrer. Thus, the sodium alginate/cornstarch solution was mixed with IMS, C1, C2, or C3 solutions under mild agitation (250 rpm) for 30 min. Hence, each solution was added drop-wise on the CaCl_2_ solution through a fine stainless-steel needle (internal diameter tip: 0.8 mm), and then, the beads were maintained in the CaCl_2_ solution for 30 min, and later, the CSAlg hydrogel beads filled with IMS, C1, C2, and C3 solutions and the Alg hydrogel beads filled with IMS, C1, C2, and C3 solutions were filtered and subsequently washed with distilled water (Figure 9).

### 4.4. Efficiency of Encapsulation (EE%)

EE% was established as the GA concentration encapsulated into CSAlg or Alg beads in relation to the initial GA concentration. The determination of GA was made after the dissolution of the beads in a Na_3_C_6_H_5_O_7_(H_2_O)_2_ solution (2% *w*/*v*) at 37 ± 2 °C. Briefly, the fresh beads (0.5 g) were homogenized with 3.5 mL of Na_3_C_6_H_5_O_7_(H_2_O)_2_ solution for 15 min using a vortex mixer (ZX3, Velp Scientifica, Usmate, Italy) [17]. In the next step, the supernatant was filter and the GA in GA–Na_3_C_6_H_5_O_7_(H_2_O)_2_ solution was determined spectrophotometrically using the Folin–Ciocalteu method [64]. The results were expressed as mg GAE/L. All samples were analyzed in triplicate. The EE% was calculated using Equation (1).

(1) $$EE\left(\%\right)=\left(\frac{L}{L_{o}}\right)*100$$

where L and L_0_ are the GA concentration released from the beads and the initial GA concentration (theoretical load), respectively.

### 4.5. Rheological Measurements

The rheological behavior of the Alg hydrogels solutions mixed with IMS, C1, C2, or C3 solutions and the CSAlg hydrogels solutions mixed with IMS, C1, C2, or C3 solutions was determined using a strain- and stress-controlled rheometer (Physica MCR300, Anton Paar GmbH, Stuttgart, Germany) at room temperature (25 °C). The instrument was equipped with smooth parallel plate geometry (50 mm diameter), and the gap between the plates was 0.8 mm. The test temperature was kept constant at 25 °C and was controlled internally by a Peltier plate system coupled to a water circulation unit. The shear rate varied between 0.1 and 100 s^−1^ to determine the flow behavior of the encapsulated solutions. The curves were fitted with Newtonian, Power Law, and Herschel–Bulkley models. The viscoelastic properties of the encapsulated solutions were determined by an oscillating frequency sweep from 0.1 to 100 Hz at a constant strain of 1% [65]. The values of the storage modulus (G′) and loss modulus (G″) were recorded with the US200 Physica software, version 2.01. All samples were analyzed in triplicate.

(2) $$\mathrm{Newtonian}\;\mathrm{model},\;\sigma =K\times \dot{\gamma }$$

(3) $$\mathrm{Power}\;\mathrm{Law}\;\mathrm{model},\;\sigma =K\times {\dot{\gamma }}^{n}$$

(4) $$\mathrm{Herschel–Bulkley}\;\mathrm{model},\;\sigma =\sigma_{0}+K\times {\dot{\gamma }}^{n}$$

where σ is the shear stress exerted by the fluid (Pa), K is the consistency index (Pa·s^n^), γ is the shear rate, η is the flow behavior index (dimensionless), and σ_0_ is the initial shear stress (Pa).

### 4.6. FTIR-ATR Measurements

Infrared spectra were recorded with a FTIR-ATR spectrometer (IRPrestige-21, Shimadzu Corporation, Kyoto, Japan). The samples were ground, and approximately 2 mg of sample was placed on a ZnSe crystal with a helium/neon laser. The data were collected in a range of 400–4000 cm^−1^, with a resolution of 4 cm^−1^ and 64 scans [66], and the results were obtained by IRSolution software, version 1.60. The ambient air was used as background. All samples were analyzed in triplicate.

### 4.7. Measurements of Thermal Properties 

Differential scanning calorimetry (DSC) analysis was conducted using a DSC apparatus (DSC1, Mettler Toledo, Schwerzenbach, Switzerland). Approximately 10 mg of whole hydrogel bead sample was placed in an aluminum crucible, and an empty aluminum crucible was used as a reference. The samples were heated from 25 to 300 °C at a heating rate of 10 °C/min in a high-purity nitrogen atmosphere at 50 mL/min [52]. The thermal properties of the samples were obtained by Stareware software, version 10.01. All samples were analyzed in triplicate.

### 4.8. Simulated Gastric Digestion (SGD)

SGD of the hydrogel beads was carried out according to the international consensus method to simulate gastrointestinal digestion (only gastric digestion), i.e., the standardized static in vitro digestion protocol by the INFOGEST^®^ network [67], with slight modifications. For the oral phase, the hydrogel beads (10 g) were placed in amber flasks and mixed with simulated salivary fluid (pH 7.0) (1:1 (*w*/*w*)), containing amylase (75 U/mL), and then, the mixture was incubated for 2 min at 37 °C, with constant agitation (300 rpm) using an orbital shaking water bath (Memmert, D-91126, Schwabach, Germany). For the gastric phase, the bolus was mixed with simulated gastric fluid (1:1 (*v*/*v*)), containing porcine pepsin (2000 U/mL), and thus, the mixture was incubated for 2 h at 37 °C (pH 3.0, adjusted with HCl 1 M), with constant agitation (300 rpm) using an orbital shaking water bath. Therefore, for each phase digestion, aliquots of the samples were obtained for gallic acid determination. Specifically, for the oral phase, aliquots were taken at time point of 0, 1, and 2 min, while for the gastric phase, aliquots were taken at time point of 0, 5, 10, 20, 30, 60, 90, and 120 min. The enzymatic reaction was stopped with a heat-shock treatment in a boiling water bath for 15 min at −80 °C. The samples were centrifuged for 10 min at 4000 rpm at 4 ℃ to separate the supernatant. The samples were analyzed 24 h after digestion for GAE. All samples were analyzed in triplicate.

### 4.9. Release Kinetics of GA Encapsulates

The release kinetics of GA from CSAlg and Alg hydrogel beads (filled with IMS, C1, C2, and C3) under in vitro SGD were computed in the zero-order kinetic, first-order kinetic, and Korsmeyer–Peppas model functions, and these functions are presented in Equations (5)–(7), respectively [68].

(5) $$\frac{M_{t}}{M_{\infty }}=kt$$

(6) $$ln\left(\frac{{1-M}_{t}}{M_{\infty }}\right)=-kt$$

(7) $$\frac{M_{t}}{M_{\infty }}=kt^{n}$$

where M_t_ is the amount of GA released at time t (mg/mL), M_∞_ is the total amount of GA released (mg/mL), t is the release time (min), k is the kinetic constant, and n is the release exponent indicating the mechanism of GA release.

### 4.10. Statistical Analysis

The results were reported as mean ± standard deviation (SD). A one-way analysis of variance (ANOVA) was applied for each variable to identify differences between samples, and least significant difference test (LSD) tests were performed for the comparison of means at a significance level of 5% (*p* ≤ 0.05) using Statgraphics Centurion XVI Software (Statpoint Technologies Inc., Warrenton, VG, USA).

## Figures and Tables

**Table 1:** Parameters of the Newtonian model, Power Law model, and the Herschel–Bulkley model.

Samples	Newtonian Fitting Parameters			Power Law Fitting Parameters			Herschel–Bulkley Fitting Parameters			
	K	η	R^2^	K	η	R^2^	τ_0_	K	η	R^2^
Alg/IMS	0.04 ± 0.00 ^a^	0.88 ± 0.02 ^a^	0.83	0.08 ± 0.00 ^a^	0.88 ± 0.02 ^a^	0.97	0.01 ± 0.00 ^a^	0.03 ± 0.00 ^a^	0.98 ± 0.01 ^a^	0.97
Alg/C1	0.13 ± 0.05 ^b^	0.87 ± 0.01 ^a,b^	0.84	0.16 ± 0.03 ^b^	0.87 ± 0.01 ^a^	0.98	0.03 ± 0.00 ^b^	0.19 ± 0.01 ^b^	0.89 ± 0.07 ^b^	0.99
Alg/C2	0.69 ± 0.04 ^c^	0.85 ± 0.04 ^a,b^	0.69	0.94 ± 0.06 ^c^	0.85 ± 0.04 ^a,b^	0.99	0.12 ± 0.01 ^c^	0.99 ± 0.01 ^c^	0.83 ± 0.06 ^b,c^	0.99
Alg/C3	1.78 ± 0.24 ^d^	0.73 ± 0.04 ^c^	0.69	3.88 ± 0.71 ^d^	0.73 ± 0.04 ^c^	0.99	0.47 ± 0.01 ^d^	4.99 ± 0.07 ^d^	0.64 ± 0.03 ^d^	0.99
CSAlg/IMS	0.06 ± 0.00 ^a^	0.92 ± 0.08 ^a^	0.84	0.10 ± 0.03 ^a^	0.92 ± 0.08 ^a^	0.96	0.03 ± 0.00 ^a^	0.03 ± 0.00 ^a^	0.98 ± 0.08 ^a^	0.96
CSAlg/C1	0.23 ± 0.02 ^b^	0.84 ± 0.05 ^a,b^	0.84	0.28 ± 0.00 ^b^	0.84 ± 0.05 ^a,b^	0.99	0.03 ± 0.00 ^a^	0.25 ± 0.01 ^b^	0.94 ± 0.07 ^a,b^	0.99
CSAlg/C2	0.81 ± 0.13 ^c^	0.78 ± 0.03 ^b,c^	0.80	1.18 ± 0.44 ^c^	0.78 ± 0.03 ^b,c^	0.99	0.17 ± 0.01 ^b^	1.27 ± 0.04 ^c^	0.81 ± 0.09 ^b,c^	0.99
CSAlg/C3	1.91 ± 0.16 ^d^	0.75 ± 0.01 ^c,d^	0.69	3.89 ± 0.64 ^d^	0.75 ± 0.01 ^c,d^	0.99	0.47 ± 0.01 ^c^	4.95 ± 0.03 ^d^	0.66 ± 0.08 ^d^	0.99

The data in the table are the mean ± SD of triplicate. Different letters in the same column indicate significant differences (*p* ≤ 0.05) by one-way ANOVA and LSD test. Alg: sodium alginate solution; CSAlg: corn starch sodium alginate solution; IMS: initial model solution; C1: concentrate from cycle 1; C2: concentrate from cycle 2; C3: concentrate from cycle 3; K: consistency index; η: flow behavior index; R^2^: coefficient of determination; τ_0_: yield stress.

**Table 2:** Thermodynamic parameters of hydrogel beads.

Sample	Peak 1		Peak 2	
	T (°C)	ΔH (mJ/g)	T (°C)	ΔH (mJ/g)
Alg/IMS	95.32 ± 12.46 ^a^	142.29 ± 20.51 ^a^	165.79 ± 23.54 ^a^	3501.22 ± 123.24 ^g^
Alg/C1	-	-	184.99 ± 14.61 ^e^	3225.03 ± 142.61 ^f^
Alg/C2	-	-	177.88 ± 32.58 ^c^	3837.35 ± 108.22 ^h^
Alg/C3	-	-	176.84 ± 46.21 ^c^	2994.73 ± 205.41 ^d^
CSAlg/IMS	96.13 ± 11.86 ^a^	508.28 ± 72.01 ^b^	174.26 ± 23.61 ^b^	466.64 ± 75.14 ^a^
CSAlg/C1	-	-	185.41 ± 12.72 ^e,f^	2717.01 ± 102.66 ^c^
CSAlg/C2	-	-	187.58 ± 34.27 ^f^	3149.71 ± 134.22 ^e^
CSAlg/C3	-	-	180.69 ± 28.98 ^d^	2433.92 ± 156.78 ^b^

The data in the table are the mean ± SD of triplicate. Different letters in the same column indicate significant differences (*p* ≤ 0.05) by one-way ANOVA and LSD test. Alg: sodium alginate solution; CSAlg: corn starch sodium alginate solution; IMS: initial model solution; C1: concentrate from cycle 1; C2: concentrate from cycle 2; C3: concentrate from cycle 3.

**Table 3:** Gallic acid solution released under Korsmeyer–Peppas model.

Samples	Korsmeyer–Peppas Model		
	k	n	R^2^
Alg/IMS	929.18 ± 23.08 ^a^	0.21 ± 0.03 ^a^	0.96
Alg/C1	1066.37 ± 15.98 ^b^	0.16 ± 0.00 ^b^	0.96
Alg/C2	1109.45 ± 53.45 ^b,c^	0.16 ± 0.02 ^b^	0.96
Alg/C3	2330.11 ± 21.00 ^d^	0.14 ± 0.01 ^b,c^	0.88
CSAlg/IMS	289.50 ± 35.68 ^a^	0.32 ± 0.04 ^a^	0.96
CSAlg/C1	771.44 ± 16.83 ^b^	0.27 ± 0.00 ^b^	0.99
CSAlg/C2	846.99 ± 0.00 ^c^	0.24 ± 0.05 ^b^	0.97
CSAlg/C3	1201.79 ± 10.16 ^d^	0.17 ± 0.00 ^c^	0.95

The data in the table are the mean ± SD of triplicate. Different letters in the same column indicate significant differences (*p* ≤ 0.05) by one-way ANOVA and LSD test. Alg: sodium alginate solution; CSAlg: corn starch- sodium alginate solution; IMS: initial model solution; C1: concentrate from cycle 1; C2: concentrate from cycle 2; C3: concentrate from cycle 3; k: kinetic constant; n: release exponent; R^2^: coefficient of determination.

## Data Availability

The data presented in this study are available on request from the corresponding authors.

## Supplementary Materials

The following supporting information can be downloaded at: https://www.mdpi.com/article/10.3390/gels9050374/s1, Table S1. Data of apparent viscosity versus shear rate for Figure 3; Table S2. Storage (G′) and loss (G″) moduli of hydrogel solutions for Figure 5a; Table S3. Storage (G′) and loss (G″) moduli of hydrogel solutions for Figure 5b.
