# Supplementary material for: Encapsulation of Concentrated Solution Obtained by Block Freeze Concentration in Calcium Alginate and Corn Starch Calcium Alginate Hydrogel Beads
Source: Gels. 2023 May 1;9(5):374. doi: 10.3390/gels9050374 (PMC10217800; doi:10.3390/gels9050374)
Supplement: Supplementary file 1 [file gels-09-00374-s001.zip › gels-2307883-supplementary.pdf]

Article

Encapsulation of concentrated solution obtained by block freeze concentration in calcium alginate and corn starch calcium alginate hydrogel beads

Patricio Orellana-Palma [1,*], Loren Macias-Bu [2], Nailín Carvajal-Mena [3], Guillermo Petzold [3] and Maria Guerra-Valle [4,*]

[1] Departamento de Ingeniería en Alimentos, Facultad de Ingeniería, Campus Andrés Bello, Universidad de La Serena, Av. Raúl Bitrán 1305, La Serena 1720010, Chile
[2] Facultad de Ciencias Tecnológicas, Universidad Nacional de Agricultura, Carretera a Dulce Nombre de Culmí km 215, Barrio El Espino, Catacamas 16201, Honduras; lmacias@unag.edu.hn (L.M.-B.)
[3] Departamento de Ingeniería en Alimentos, Facultad de Ciencias de la Salud y de los Alimentos, Campus Fernando May, Universidad del Bío-Bío, Av. Andrés Bello 720, Chillán 3780000, Chile; nailin.carvajal1801@alumnos.ubiobio.cl (N.C.-M); gpetzold@ubiobio.cl (G.P.)
[4] Departamento de Nutrición y Dietética, Facultad de Ciencias para el Cuidado de la Salud, Campus Concepción, Universidad San Sebastián, Lientur 1457, Concepción 4080871, Chile
* Correspondence: patricio.orellanap@userena.cl (P.O.-P.); maria.guerra@uss.cl (M.G.-V.); Tel.: +56-52-2204304 (P.O.-P.); +56-2-27877032 (M.G.-V)

Supplementary Information

Contains: 3 Tables

Table S1. Data of apparent viscosity versus shear rate for Figure 3.

Apparent viscosity (Pa·s)	Shear rate (1/s)				Shear rate (1/s)			
	Alg/IMS	Alg/C1	Alg/C2	Alg/C3	CSAlg/IMS	CSAlg/C1	CSAlg/C2	CSAlg/C3
0,10	4,65	1,18	0,34	0,31	4,69	1,66	0,41	0,34
0,13	5,31	1,24	0,41	0,25	5,50	1,57	0,34	0,39
0,16	5,55	1,20	0,36	0,12	5,78	1,51	0,40	0,33
0,20	5,57	1,17	0,32	0,09	5,87	1,40	0,37	0,27
0,26	5,53	1,18	0,23	0,09	5,82	1,34	0,40	0,19
0,33	5,45	1,18	0,17	0,11	5,69	1,32	0,38	0,12
0,42	5,37	1,12	0,13	0,11	5,51	1,36	0,30	0,11
0,53	5,13	1,02	0,17	0,03	5,34	1,31	0,25	0,13
0,67	4,89	1,01	0,12	0,04	5,01	1,29	0,29	0,08
0,85	4,63	0,97	0,11	0,02	4,66	1,29	0,25	0,09
1,08	4,28	0,94	0,09	0,04	4,31	1,23	0,25	0,04
1,37	3,93	0,89	0,12	0,05	3,99	1,14	0,23	0,04
1,74	3,61	0,85	0,14	0,07	3,70	1,11	0,22	0,05
2,21	3,35	0,86	0,14	0,08	3,45	1,20	0,25	0,07
2,81	3,13	0,84	0,13	0,06	3,24	1,06	0,24	0,07
3,56	2,89	0,80	0,12	0,06	3,02	1,01	0,23	0,05
4,52	2,67	0,79	0,13	0,07	2,81	0,99	0,24	0,07
5,74	2,48	0,75	0,12	0,06	2,62	0,94	0,23	0,06
7,28	2,30	0,74	0,13	0,06	2,44	0,89	0,23	0,06
9,24	2,14	0,71	0,13	0,06	2,28	0,86	0,23	0,07
11,70	1,98	0,68	0,13	0,06	2,11	0,81	0,23	0,06
14,90	1,84	0,65	0,13	0,06	1,96	0,77	0,22	0,06
18,90	1,71	0,63	0,13	0,06	1,81	0,73	0,22	0,06
24,00	1,58	0,60	0,12	0,06	1,69	0,70	0,22	0,06
30,40	1,47	0,57	0,12	0,06	1,56	0,66	0,21	0,06
38,60	1,37	0,55	0,12	0,06	1,45	0,63	0,21	0,06
48,90	1,27	0,52	0,12	0,06	1,36	0,60	0,20	0,06
62,10	1,18	0,50	0,12	0,06	1,26	0,56	0,20	0,06
78,80	1,09	0,47	0,12	0,06	1,17	0,53	0,19	0,06
100,00	1,02	0,45	0,11	0,06	1,09	0,50	0,19	0,06

Alg: sodium alginate solution, CSAlg: corn starch sodium alginate solution, IMS: initial model solution, C1: concentrate from cycle 1, C2: concentrate from cycle 2, C3: concentrate from cycle 3.

Table S2. Storage (G′) and loss (G′′) moduli of hydrogel solutions for Figure 5a.

Angular frequency (rad/s)	Alg/IMS		Alg/C1		Alg/C2		Alg/C3	
	G′	G′′	G′	G′′	G′	G′′	G′	G′′
0,1	0,0000043	0,00831	0,00000222	0,0161	0,0000134	0,0794	3,28E-05	0,139
3,54	0,0000059	0,464	0,00000336	0,778	0,0000149	1,53	0,0000343	3,02
6,99	0,0000126	0,503	0,00000642	1,02	1,61E-05	2,45	0,0388	5,35
10,4	0,0000167	0,776	0,00000652	1,56	1,68E-05	3,38	0,942	7,41
13,9	0,0000181	1,15	0,00000671	2	0,0000186	4,28	1,42	9,42
17,3	0,00000521	1,57	0,00000683	2,4	0,0000195	5,05	1,63	11,2
20,8	0,00171	1,8	0,00000745	3,03	0,0000234	5,94	2,29	12,7
24,2	0,0162	1,84	0,00000754	3,63	0,0000243	6,73	2,72	14,8
27,7	0,0459	1,87	0,00000808	3,85	0,0000256	7,61	3,32	15,8
31,1	0,0529	2,61	0,00000838	4,29	0,0000279	8,42	3,52	18,5
34,5	0,101	3,36	0,00000842	4,77	0,0000294	9,04	3,89	19,5
38	0,242	3,65	0,00000874	4,83	0,0000302	9,81	3,95	20,4
41,4	0,986	3,9	0,00000937	5,55	0,0000317	10,3	4,77	22,3
44,9	1,21	3,93	0,00000949	6,16	0,0608	11,4	5,23	24,3
48,3	1,6	4,58	0,0000102	6,63	0,6	11,8	5,69	25,8
51,8	1,87	4,64	0,0000106	6,93	0,795	12,1	6	26,7
55,2	3,11	4,71	0,000011	7,04	0,974	13	7,72	27,6
58,7	3,37	5,12	0,0029	7,24	1,08	14,4	8,35	29,6
62,1	3,54	5,15	0,0678	7,37	1,19	16,1	10,7	30,4
65,6	3,76	5,26	0,0715	8,03	1,34	17,3	11,1	31,5
69	4,05	5,62	0,0844	8,13	1,48	18,1	12,1	35,2
72,4	4,17	5,95	0,293	8,72	1,49	20,1	12,6	35,4
75,9	4,75	6,03	0,32	9,05	1,57	21	12,6	36,9
79,3	4,78	6,34	0,596	9,09	1,64	25,3	12,7	37
82,8	6,27	6,36	0,606	9,43	1,76	26,3	13	37,7
86,2	6,84	6,52	0,643	10,1	2,9	27,6	13,7	38,8
89,7	8,96	7,25	1,65	10,2	4,11	30,1	14,2	40,1
93,1	9,6	8,1	2,88	11	4,39	31,8	17,9	41,5
96,6	10,12	8,62	3,68	11,4	4,79	32,6	18,2	41,9
100	11,68	9,35	4,19	11,9	5,7	34,2	20	44,2

Alg: sodium alginate solution, IMS: initial model solution, C1: concentrate from cycle 1, C2: concentrate from cycle 2, C3: concentrate from cycle 3.

Table S3. Storage (G′) and loss (G′′) moduli of hydrogel solutions for Figure 5b.

Angular frequency (rad/s)	CSAlg/IMS		CSAlg/C1		CSAlg/C2		CSAlg/C3	
	G′	G′′	G′	G′′	G′	G′′	G′	G′′
0,1	0,000000512	0,00787	0,00000257	0,0173	0,0000179	0,0667	3,42E-05	0,21
3,54	0,00000187	0,484	0,00000349	0,706	0,0000199	1,86	0,0000358	3,91
6,99	0,00000223	0,552	0,00000349	0,768	0,00313	3,51	0,0000388	6,88
10,4	0,00000271	0,72	0,00000366	1,16	0,138	4,89	0,0000422	9,49
13,9	0,00000316	0,991	0,00000551	1,53	0,314	6,47	0,0000464	11,9
17,3	0,00000402	1,26	0,00000585	1,85	0,373	7,84	0,0000466	14,3
20,8	0,00000509	1,87	0,0000063	2,25	0,666	9,17	0,0000479	16,6
24,2	0,00000537	2,02	0,00000769	2,78	0,778	10,5	0,0000499	19,2
27,7	0,00000589	2,32	0,00000795	3,77	0,779	11,5	0,0000524	21,6
31,1	0,00000601	2,41	0,0000117	3,76	0,904	12,7	0,000041	23,8
34,5	0,00000828	2,93	0,0000299	3,95	1,03	13,4	0,000044	26,8
38	0,00000834	3,41	0,0104	5,16	1,2	15,1	0,018	29,7
41,4	0,00000946	3,47	0,153	5,72	2,71	16,7	1,2	31,5
44,9	0,0000097	4,06	0,158	5,94	2,84	17,6	2,42	33,9
48,3	0,0000103	4,34	0,19	5,99	4,41	18,3	2,9	36,9
51,8	0,0000211	4,49	0,311	6,31	4,47	19,3	3,8	38,6
55,2	0,000679	4,68	0,324	6,39	4,76	20,4	4,95	41,9
58,7	0,108	4,75	0,4	6,8	5,65	21,4	5,01	44,3
62,1	0,124	5,37	0,499	7	5,76	22,3	5,38	45,6
65,6	0,17	5,49	1,13	7,13	6,32	23,2	6,12	47,5
69	0,192	5,57	1,46	7,22	6,42	24,1	6,68	50,1
72,4	0,197	5,79	2,01	7,8	6,45	25,2	7,03	50,3
75,9	0,685	6,35	2,99	7,88	6,81	25,8	8,19	51,6
79,3	0,812	6,48	3,01	8,3	7,27	27	8,42	53,9
82,8	1,24	8,93	3,6	8,33	7,34	27,9	8,87	53,9
86,2	1,46	9	4,84	8,58	7,39	28,7	9,29	56,5
89,7	2,05	10,2	5	8,97	7,4	30,5	9,62	57,4
93,1	3,1	10,5	7,7	10,1	7,84	31,5	10,9	59
96,6	4	11,2	8,6	12,6	8,32	32,1	13,5	61,4
100	4,4	12,8	10,3	14,2	8,42	32,8	15	62,5

CSAlg: corn starch sodium alginate solution, IMS: initial model solution, C1: concentrate from cycle 1, C2: concentrate from cycle 2, C3: concentrate from cycle 3.
